# Supplementary material for: Ionised and total hypocalcaemia in pregnancy: An analysis of prevalence and risk factors in a resource-limited setting, Cameroon
Source: PLoS One. 2022 May 18;17(5):e0268643. doi: 10.1371/journal.pone.0268643 (PMC9116680; doi:10.1371/journal.pone.0268643)
Supplement: Attachment — Submitted filename: Response to reviewers.docx [file pone.0268643.s002.docx]

**RESPONSE TO REVIEWER’S COMMENTS**

| **General reviewers comments** | **Response to reviewers comments** |
| --- | --- |
|  | We wish to appreciate the efforts and work done by the reviewer to strengthen this manuscript. The comments raised by the reviewer have gone a long way to significantly improve the quality of the manuscript. The response to the different reviewer’s comments are presented below. |
| This manuscript explores associations of low blood Ca in pregnant women and various sociodemographic, obstetric or nutritional factors. This cross-sectional study was conducted in 4 different health care settings in Cameroon. | Thanks for the observation. |
| Consider using the term low blood Ca in clinically healthy subject throughout the manuscript, instead of hypocalcemia. Please note that the title reads low blood Ca. Hypocalcemia connotates an abnormality with potentially negative clinical effects. | Thanks for your suggestion. Hypocalcaemia (generally used to mean “lower than normal blood calcium levels”) can either be an asymptomatic laboratory finding or a life-threatening metabolic disturbance (1)(2).  We however agree on the precision “in clinically healthy or “apparently” healthy pregnant women” which has been used throughout the manuscript and made clear in the conclusion. This correction has been integrated in the whole manuscript where necessary. |
| No reference has been cited to support the choice of low blood Ca/hypocalcemia thresholds. | Thanks for the observations. The reference has been included. See page 10, first paragraph |
| There is a large disagreement in prevalence of low blood Ca, based on the type of the analytical method of choice. This issue deserves further attention. Please consider the following: 1. Visually evaluate your data by plotting iCa (consider the gold standard) vs total calcium, and iCa vs albumin-corrected total Ca. 2. Evaluate the correlation of iCa with tCa and albumin-corrected tCa. 3. Consider evaluating if there is a bias across lab methods, and if this bias is constant. | Thank you for your observations and suggestions. These were some original worries when the protocol was been designed. We intended to use ionised hypocalcaemia as a gold standard to evaluate the sensitivity of total calcaemia measurements in defining hypocalcaemia. We however found out that this was not scientifically correct given the ionised and total calcium are not exactly the same laboratory finding diagnosed by different methods. Multiple studies have agreed that even though total hypocalcaemia seems highly prevalent in pregnancy (laboratory finding), this constitutes some form of false or factitious hypocalcaemia associated with physiological changes in pregnancy that can be ignored (3). Ionised and total hypocalcaemia in pregnancy should therefore be studied as two entities as they might be associated with adverse outcomes of different severities.  However, the results of the requested additional analyses have been presented in the results section. See the two attached figures and page 14, paragraph 2 and page 15  Given that we are measuring different outcomes with different methods, we think that it will not make sense to assess bias across methods. Besides some studies suggest that different health conditions affect these different forms of calcium differently. |
| The criterion used to stratify the sociodemographic (i.e., age, monthly revenue, household occupants), obstetric (i.e., gestational age at first antenatal visit, number of visits, number of pregnancies, number of deliveries), and nutritional factors (i.e., taking fruit rare) should be clear. Describe the reasoning behind the choice of categories | Thank you for your question. Given that we did not find any similar studies carried out with categorization. Most of the categorization was done from previous studies carried out in pregnancy and particular WHO health indicators. Some of the variables were categorized following standard divisions like in a union/not in a union.  Concerning fruit consumption, people who consumed fruits rarely were expected to have hypocalcaemia compared with their counterparts who do more often (4).  See paragraph 2, page 10. |
| Authors do not provide enough background to understand the selected nutritional factors as explanatory variables within the context of Cameroon dietarian preferences. For example, it is unclear why authors decided on oranges, watermelons, and pineapples to classify the most consumed fruit. “Eating fruit rarely” it is not a quantifiable variable and may have different meaning across countries or ethnicities (i.e., number of pieces or servings per day). | Thanks for your suggestion. Studies around nutritional habits and practices in Cameroon are still very few. Studies carried out in pregnant women in Cameroon are ever rarer. We did not have any published studies on dietarian practices.  Concerning fruits, Consumption of vegetables and fruits has been reported to better calcium availability in blood and bone mineral density. We therefore believed that women who consumed fruits rarely were likely to have lower calcaemia compared to their counterparts who consumed fruits more often (4).  We recognize the fact that this variable is not quantifiable and will include this in the limits of the study. In our context, it is really difficult to evaluate this in pieces or servings per day). See limits section, page 30, paragraph 2.  As concerns the most consumed fruits, only pineapples, oranges, watermelons and ripe banana were specified in the questionnaire and all other fruits included into others. When analysed without categories, no particular fruit had a statistically significant association with hypocalcaemia. As stated in the background, the most consumed fruits were watermelon, oranges and pineapples. We therefore wanted to know if consumption of the most frequently consumed fruits in Cameroon was associated with hypocalcaemia. Some information on the nutritional behaviors has been added to the background. See page 5, paragraph 3. |

| **Specific Reviewers comments** | **Response to comments** |
| --- | --- |
| L 1. Acknowledge that the study was conducted in Cameroon. | Thanks for the recommendation. Cameroon has been added to the title. See line 1 |
| L164. As indicated above, authors need to support the hypocalcemia thresholds with references (iCa, albumin-corrected tCa, and tCa). | Thanks for your recommendations, the references have been added. Already addressed above |
| L 164. Report the number of cows identified with low Ca serum when combining result from the 3 methods. | Thank you for the recommendation. The number of women meeting the criteria has been added. Only 28 participants met this criteria. See results section, page 14, paragraph 1. |
| L 202. Please explain in materials how data was analyzed to calculate the prevalence of low calcium in blood. | Thank you for the suggestion. The cut-offs considered were stated clearly in the data analysis section. All women who had ionised, total crude and albumin-corrected total calcium levels less than or equal to the stated cut-offs were classified as hypocalcaemic (low blood calcium). This statement has been included in the data analysis section. See page 10, paragraph 1. |
| L 217. Consider rephrasing as “Sociodemographic and obstetric factors associated with hypocalcemia are presented in Table 3.” | Thank you for your correction. Adopted in the text. See page 16, paragraph 1. |
| L 236. Change “Table 8: Nutritional factors affected” by “Table 4: Nutritional factors associated”. | Thank you for the correction. Corrected accordingly. See table 4, page 19. |
| L 250. The first discussion paragraph is written as results. Follow scientific writing guidelines for discussion, and delete it. | Thank you for your recommendation. It has been deleted and reformulated. See paragraph 1, page 21. |
| L 250. It is striking the difference in prevalence when considering different lab methods. That should be one of the first points of discussion. | Thanks for the suggestion. The discussion has been shifted up to start by discussing the discrepancy. The laboratory methods can really not be discussed here given that these are recommended and gold standard procedures especially for ISE potentiometry. The methods adopted (AAS and ISEP) measure two different targets (ionised calcium and total calcium). Ideally ionised (physiological) hypocalcaemia should be the true target and not the total hypocalcaemia, even though having total hypocalcaemia increased the chances of having ionised hypocalcaemia (stated in the discussion). See page 27, paragraph 2. As stated above additional analysis has been presented. |
| L 260 to 262. Alkalogenic diets (rick in K) may induce pseudohypoparathyroidism. Please see http://doi.org/10.3618/jds.2013-7467 | Thanks for your suggestion. This has been included in the discussion. See page page 28, last paragraph. |
| L 265. Replace “borderline significance” by “tendency to”. Define in material and methods tendency, for example p< 0.1 | Thanks for the suggestion. We prefer to state clearly that the association was not statistically significant. And include the tendency in the next sentence. See page 30, paragraph 1. |
| L 280. What was the prevalence with unadjusted Calcium levels? | Thank you for the question. The prevalence with unadjusted calcium levels is the prevalence of totalcrude hypocalcaemia. That is, when the total calcium levels are not adjusted or corrected for albumin changes. This has been reported in the results and first paragragh of the discussion. The word “unadjusted” has been changed and made more explicit. See page 24, paragraph 2. |
| L 296 to 299. Disagreement in Ca concentration across lab methods has been reported in patients with blood Ca metabolism disorders. However, the present study included healthy patients. Are there other plausible explanations? As indicated above, please explore the data. | Thanks for your concern. As already explained above, only two diagnostic methods were used and these two diagnostic methods did not measure the same calcium fractions in blood. One measured and classified calcium levels based on the physiologically active fraction which is the absolute hypocalcaemia, while the other method measured the whole calcium in circulation (which can be considered a relative hypocalcaemia). See page 21, paragraph 2, sentence 3 and 4, of discussion |
| L 306. Replace regnancies with pregnancies. | Thank you for the correction. This has been corrected. See page 22, first paragraph. |
| L 320. This section of the discussion focuses on VitD, but it was not measured in this study. L 328. Do no start a new paragraph with Therefore. | Thanks for the observation. Vit D deficiency and other hormonal disorders were not measured in this study (stated as a limit). The discussion on these was just to identify based on relative prevalence from other studies, the plausible causes of the hypocalcaemia (particularly the ionised hypocalcaemia) in pregnancy.  The “therefore” has been taken off the sentence. See page 23, paragraph 2 |
| L 401. Another limitation of the study is that the physiological implications of low blood calcium were not explored. | Thanks for the suggestion. This has been included as a limit. See page 30, last paragragh. |
| Tables Remove the table’s superscripts indicating statistical significance (no needed). Explain in tables and manuscript if all the variables selected to be in multivariable model based on p-value, remained in the model or were removed after backward elimination. The tables should stand on their own. Include the definition of hypocalcemia (lab method and threshold) used in the data analysis. | Thanks for the recommendations. The tables have been edited accordingly. See tables 3, 4 and 5. |

| **Specific Reviewers comments** | **Response to comments** |
| --- | --- |
| L 1. Acknowledge that the study was conducted in Cameroon. | Thanks for the recommendation. Cameroon has been added to the title. See line 1 |
| L164. As indicated above, authors need to support the hypocalcemia thresholds with references (iCa, albumin-corrected tCa, and tCa). | Thanks for your recommendations, the references have been added. Already addressed above |
| L 164. Report the number of cows identified with low Ca serum when combining result from the 3 methods. | Thank you for the recommendation. The number of women meeting the criteria has been added. Only 28 participants met this criteria. See results section, page 14, paragraph 1. |
| L 202. Please explain in materials how data was analyzed to calculate the prevalence of low calcium in blood. | Thank you for the suggestion. The cut-offs considered were stated clearly in the data analysis section. All women who had ionised, total crude and albumin-corrected total calcium levels less than or equal to the stated cut-offs were classified as hypocalcaemic (low blood calcium). This statement has been included in the data analysis section. See page 10, paragraph 1. |
| L 217. Consider rephrasing as “Sociodemographic and obstetric factors associated with hypocalcemia are presented in Table 3.” | Thank you for your correction. Adopted in the text. See page 16, paragraph 1. |
| L 236. Change “Table 8: Nutritional factors affected” by “Table 4: Nutritional factors associated”. | Thank you for the correction. Corrected accordingly. See table 4, page 19. |
| L 250. The first discussion paragraph is written as results. Follow scientific writing guidelines for discussion, and delete it. | Thank you for your recommendation. It has been deleted and reformulated. See paragraph 1, page 21. |
| L 250. It is striking the difference in prevalence when considering different lab methods. That should be one of the first points of discussion. | Thanks for the suggestion. The discussion has been shifted up to start by discussing the discrepancy. The laboratory methods can really not be discussed here given that these are recommended and gold standard procedures especially for ISE potentiometry. The methods adopted (AAS and ISEP) measure two different targets (ionised calcium and total calcium). Ideally ionised (physiological) hypocalcaemia should be the true target and not the total hypocalcaemia, even though having total hypocalcaemia increased the chances of having ionised hypocalcaemia (stated in the discussion). See page 27, paragraph 2. As stated above additional analysis has been presented. |
| L 260 to 262. Alkalogenic diets (rick in K) may induce pseudohypoparathyroidism. Please see http://doi.org/10.3618/jds.2013-7467 | Thanks for your suggestion. This has been included in the discussion. See page page 28, last paragraph. |
| L 265. Replace “borderline significance” by “tendency to”. Define in material and methods tendency, for example p< 0.1 | Thanks for the suggestion. We prefer to state clearly that the association was not statistically significant. And include the tendency in the next sentence. See page 30, paragraph 1. |
| L 280. What was the prevalence with unadjusted Calcium levels? | Thank you for the question. The prevalence with unadjusted calcium levels is the prevalence of totalcrude hypocalcaemia. That is, when the total calcium levels are not adjusted or corrected for albumin changes. This has been reported in the results and first paragragh of the discussion. The word “unadjusted” has been changed and made more explicit. See page 24, paragraph 2. |
| L 296 to 299. Disagreement in Ca concentration across lab methods has been reported in patients with blood Ca metabolism disorders. However, the present study included healthy patients. Are there other plausible explanations? As indicated above, please explore the data. | Thanks for your concern. As already explained above, only two diagnostic methods were used and these two diagnostic methods did not measure the same calcium fractions in blood. One measured and classified calcium levels based on the physiologically active fraction which is the absolute hypocalcaemia, while the other method measured the whole calcium in circulation (which can be considered a relative hypocalcaemia). See page 21, paragraph 2, sentence 3 and 4, of discussion |
| L 306. Replace regnancies with pregnancies. | Thank you for the correction. This has been corrected. See page 22, first paragraph. |
| L 320. This section of the discussion focuses on VitD, but it was not measured in this study. L 328. Do no start a new paragraph with Therefore. | Thanks for the observation. Vit D deficiency and other hormonal disorders were not measured in this study (stated as a limit). The discussion on these was just to identify based on relative prevalence from other studies, the plausible causes of the hypocalcaemia (particularly the ionised hypocalcaemia) in pregnancy.  The “therefore” has been taken off the sentence. See page 23, paragraph 2 |
| L 401. Another limitation of the study is that the physiological implications of low blood calcium were not explored. | Thanks for the suggestion. This has been included as a limit. See page 30, last paragragh. |
| Tables Remove the table’s superscripts indicating statistical significance (no needed). Explain in tables and manuscript if all the variables selected to be in multivariable model based on p-value, remained in the model or were removed after backward elimination. The tables should stand on their own. Include the definition of hypocalcemia (lab method and threshold) used in the data analysis. | Thanks for the recommendations. The tables have been edited accordingly. See tables 3, 4 and 5. |

References

1.	Pepe J, Colangelo L, Biamonte F, Sonato C, Danese VC, Cecchetti V, et al. Diagnosis and management of hypocalcemia. Endocrine. 2020;69(3):485–95.

2.	Cooper MS, Gittoes NJL. Diagnosis and management of hypocalcaemia. Bmj. 2008;336(7656):1298–302.

3.	Almaghamsi A, Almalki MH, Buhary BM. Hypocalcemia in pregnancy: A clinical review update. Oman Med J. 2018;33(6):453–62.

4.	Qiu R, Cao WT, Tian HY, He J, Chen GD, Chen YM. Greater intake of fruit and vegetables is associated with greater bone mineral density and lower osteoporosis risk in middle-aged and elderly adults. PLoS One. 2017;12(1).
