# Supplementary material for: Ionised and total hypocalcaemia in pregnancy: An analysis of prevalence and risk factors in a resource-limited setting, Cameroon
Source: PLoS One. 2022 May 18;17(5):e0268643. doi: 10.1371/journal.pone.0268643 (PMC9116680; doi:10.1371/journal.pone.0268643)
Supplement: Attachment — Submitted filename: RESPONSE TO REVIEWERS.docx [file pone.0268643.s003.docx]

**RESPONSE TO REVIEWERS**

We wish to thank the editor and reviewer for the time taken to improve on this manuscript. We have now taken time to address all the editor and reviewer comments. This is presented below.

**Editor’s comments**

**Comment**: Please review your reference list to ensure that it is complete and correct. If you have cited papers that have been retracted, please include the rationale for doing so in the manuscript text, or remove these references and replace them with relevant current references. Any changes to the reference list should be mentioned in the rebuttal letter that accompanies your revised manuscript. If you need to cite a retracted article, indicate the article’s retracted status in the References list and also include a citation and full reference for the retraction notice.

**Response**: Thanks for your advice. The references have been checked and some changes made. Ref number 8 and 11 have been changed, and one of the references which was a repetition of reference number 3 taken off. See reference list.

**Reviewer’s comments**

**Comment**: Thanks to the authors for revising the manuscript. It has greatly improved. I have additional comments that may help improve the quality of this work.

**Response**: Thanks for your observation. The efforts put in by the reviewer to improve this work are highly appreciated on our side. We must agree that the review process has highly improved on the quality of this manuscript.

**Comment**: Consider that Plos One does not copyedit manuscripts. The language used is correct but not very elegant. I will strongly recommend a professional editor to polish the manuscript prior to publication.

**Response**: Thanks for the observation. We had our manuscript screened by a native English speaker and hope the level of English is up to standard now.

**Major comment**: Why the study focuses on hypocalcemia based on total Ca (tCa) instead of ionized Ca (iCa)? Should be more appropriate to present associations with both iCa and tCa? The number of subjects with low iCa is limited but enough to explore possible associations.

**Response**: We highly appreciate the reviewers question which is pertinent. We belief that leaving this out would have taken a big chunk off this work. As recommended, we have added analysis for ionised calcaemia and changes made on the whole manuscript to reflect the analyses. This has affected the objective statement, the results, discussion and conclusion. See abstract (page 2 and 3), last paragraph of introduction (page 6), results section (page 21, 22 and 24, including table 5, 6 and 7), discussion section (page 32, second paragraph and page 33, last paragraph), conclusion section, first paragraph, page 35).

**Minor** **comment**: Revise the manuscript and use concentration instead level. Be consistent.

**Response:** Thanks for your correction. The whole manuscript has been revised as recommended.

**Specific comments**:

**Comment:** L 34. Please consider rephrasing: Blood samples were collected and analyzed for serum ionised calcium concentrations and pH (ion-selective potentiometry), and for total calcium and albumin concentration (atomic absorption spectrophotometry). Sociodemographic, obstetric and nutritional data were collected through interviewer-administered questionnaire.

**Response**: Thanks for taking time to correct this. We have adopted it as recommended. See Abstract, page 2, line 35-39.

**Comment:** L57 – 66. Indicate the type of analysis (iCa or tCa) used to evaluate hypocalcemia in studies 1 to 5.

**Response**: Thanks for your suggestion. This has been added to the first paragraph of the introduction.
**Comment:** L 167. Explain in this section the software used to do the statistical analysis, and methods for regression and logistic regression.

**Response**: Thanks for your suggestion: All these are included in the data analysis section. See page 9, 10 and 11.

**Comment:** L 167. Explain in the data analysis section what measurement and definition of hypocalcemia was used to build the multivariable models.

**Response:** Thanks for your suggestion. This has been included in the data analysis section. See page 10, paragraph 2.

**Comment:** L 248 to 251. Is this information a repetition of what presented in Fig 1 and 2? If so, delete.

**Response**: Thanks for your observation. This is not a repetition but additional information not accessible on the figures. We think the information should remain.

**Comment:** L 259. Consider using the word odds when reporting results. For example “Participants who earned less than 100.000FCFA (179 USD) in a month had 0.73 lower odds to have total crude hypocalcaemia”. Revise entire manuscript. This is important because this study reports odds no likelihood or risks.

**Response:** Thank you very much for pulling our attention to that. We have revised the whole manuscript accordingly.
**Comment:** L 308 discusses de physiological implications of iCa but the study evaluates associations with tCa.

**Response**: Thanks for your observation. We have now included associations with iCa in the manuscript.
**Comment:** L 293 to 295. This is results. It does not belong in the discussion.

Thanks very much, it has been taken up to the results section. See result section, page 16, line 286-288.
**Comment:** Table 3 and 4 footnotes, modified as:
p-value <0.05; statistical significance
p-value<0.25; eligible for multiple regression analysis

**Response**: Thanks for your suggestion. This has been modified.
