# Supplementary material for: Ionised and total hypocalcaemia in pregnancy: An analysis of prevalence and risk factors in a resource-limited setting, Cameroon
Source: PLoS One. 2022 May 18;17(5):e0268643. doi: 10.1371/journal.pone.0268643 (PMC9116680; doi:10.1371/journal.pone.0268643)
Supplement: Attachment — Submitted filename: Response to Reviewers.docx [file pone.0268643.s004.docx]

**RESPONSE TO REVIEWERS**

We appreciate the immense effort of the editor and the reviewer towards strengthening this work. We have addressed the comments raised by the editor and reviewer below.

**Editor’s comment:** Please review your reference list to ensure that it is complete and correct. If you have cited papers that have been retracted, please include the rationale for doing so in the manuscript text, or remove these references and replace them with relevant current references. Any changes to the reference list should be mentioned in the rebuttal letter that accompanies your revised manuscript. If you need to cite a retracted article, indicate the article’s retracted status in the References list and also include a citation and full reference for the retraction notice.

Response: Thanks for the recommendation. The reference list had been reviewed accordingly during the last revision and the changes were specified in the rebuttal letter.

**Reviewer’s comments**

**Comment**: Thank you for your effort. Please see below some minor comments.

**Response**: Thank you again for additional comments. We are grateful for you pertinent contributions

**Comment**: L 254 to 257 Please note that your sample size is very large and results in significant differences, but the correlation coefficient is poor.

**Response**: Thank you for the observation. We have taken this precision off given that it is not part of the objectives of this manuscript. However, we feel that not all statistical observations have direct clinical or biological implications. We already stated in the manuscript that the correlation was weak (poor). See line 254-257.

**Comment**: L 262 to 263 Your sample size is very large and results in significant differences, but it may have limited biological implications.

**Response**: Thank you for the comment; given that this is not part of any of the objectives of the manuscript, the specification has been removed. See results section, line 262-267.

**Comment**: L 332. Replace “Piece of work” with “The main goal of our study was to estimate the prevalence of …”

**Response**: Thank you for your suggestion. It has been adopted. See discussion section, line 332.

**Comment**: All the figures and tables should stand on their own without the need to gather additional information from the main body of the manuscript. Add the number of subjects in the total and ionized hypocalcemia. Please revise the space around equal signs in the tables and figures.

**Response**: Thank you for your comments; we have now made the requested additions to the tables and manuscript.
